# FragariaCyc: A Metabolic Pathway Database for Woodland Strawberry *Fragaria vesca*

**DOI:** 10.3389/fpls.2016.00242

**Published:** 2016-03-04

**Authors:** Sushma Naithani, Christina M. Partipilo, Rajani Raja, Justin L. Elser, Pankaj Jaiswal

**Affiliations:** Department of Botany and Plant Pathology, Oregon State UniversityCorvallis, OR, USA

**Keywords:** FragariaCyc, *Fragaria vesca*, strawberry, plant pathway database, metabolic network, gene-expression analysis

## Abstract

FragariaCyc is a strawberry-specific cellular metabolic network based on the annotated genome sequence of *Fragaria vesca* L. ssp. *vesca*, accession Hawaii 4. It was built on the Pathway-Tools platform using MetaCyc as the reference. The experimental evidences from published literature were used for supporting/editing existing entities and for the addition of new pathways, enzymes, reactions, compounds, and small molecules in the database. To date, FragariaCyc comprises 66 super-pathways, 488 unique pathways, 2348 metabolic reactions, 3507 enzymes, and 2134 compounds. In addition to searching and browsing FragariaCyc, researchers can compare pathways across various plant metabolic networks and analyze their data using Omics Viewer tool. We view FragariaCyc as a resource for the community of researchers working with strawberry and related fruit crops. It can help understanding the regulation of overall metabolism of strawberry plant during development and in response to diseases and abiotic stresses. FragariaCyc is available online at http://pathways.cgrb.oregonstate.edu.

## Introduction

Strawberries, like other fleshy fruits, are rich in essential nutrients, vitamins, anti-oxidants, dietary fibers, and diverse set of volatile compounds—all of which offer potential benefits to human health, including protection against cancer, inflammation, coronary heart diseases, and other age-related diseases (Nijveldt et al., 2001; Boyer and Liu, 2004; Gallus et al., 2005; Liu et al., 2005; Rasmussen et al., 2005; Giampieri et al., 2015; Mazzoni et al., 2016). For that reason, strawberries have been the subject of extensive research using both chemical (Du et al., 2011; Egea et al., 2014; Prat et al., 2014; Schwieterman et al., 2014) and molecular approaches (Schwab et al., 2009; Chambers et al., 2014; Najda et al., 2014; Sanchez-Sevilla et al., 2015; Vallarino et al., 2015; Paniagua et al., 2016). In the past decade, high-throughput experimental approaches have been employed to study strawberry transcriptomes, metabolomes, and proteomes (Perez et al., 1999; Aharoni and O'connell, 2002; Wein et al., 2002; Aharoni et al., 2004; Lunkenbein et al., 2006b; Fait et al., 2008; Bombarely et al., 2010; Zhang et al., 2011). However, these omics data have not been fully utilized due to the lack of a comprehensive cellular metabolic framework for analysis.

*Fragaria vesca*, the diploid woodland strawberry, is an ancestral subgenome donor to the octoploid *F*. × *ananassa*, and its immediate ancestors *F. chiloensis* and *F. virginiana* (Rousseau-Gueutin et al., 2009). The *F. vesca* has a small stature, short life cycle, and a small sequenced genome (Shulaev et al., 2011) that is amenable to genetic manipulations (Slovin et al., 2009). It shares synteny with many commercially important fruit crop species of the family Rosaceae, such as apple, peach, pear, plum, apricot, raspberry, blueberry, etc. (Illa et al., 2011; Shulaev et al., 2011). Therefore, *F. vesca* is emerging as an attractive model for functional genomics studies within the Rosaceae family (Xia et al., 2015).

We report the development of FragariaCyc, a curated metabolic pathway database for strawberry, based on the genome sequence of the *Fragaria vesca* L. ssp. *vesca*, accession Hawaii 4 (Shulaev et al., 2011). FragariaCyc was constructed using the reference eukaryotic metabolic network MetaCyc (Caspi et al., 2014) and Pathway-Tools software (http://bioinformatics.ai.sri.com/ptools/).

Conceptually, FragariaCyc is a repertoire of the strawberry metabolic and transport pathways depicted by a complex network of dots and connectors. The metabolic reactions show the enzymatic conversion of metabolites. The enzymes with Enzyme Commission (EC) number and associated coding genes are displayed on the top of the reactions. The transport reactions portray transportation of metabolites from one subcellular compartment to another or between the cells. The pathways, reactions, genes, enzymes, and proteins carry electronic and systematic annotations based on orthology and are supported by manually curated experimental evidences. Each component has a detailed page containing a summary, citations, and other relevant information. Currently, for analyzing genomics datasets researchers are using *F. vesca* geneIDs for various members of the *Fragaria* genus. In the wider interest of the strawberry research community, we are curating FragariaCyc at the level of the genus *Fragaria*, but whenever available, we are incorporating information relevant to species or subspecies. The evidences for the existence of various metabolites, reactions, and pathways were collected from published studies on transcriptomes (Aharoni et al., 2002; Aharoni and O'connell, 2002; Folta et al., 2010; Shulaev et al., 2011), proteomes (Alm et al., 2007; Bianco et al., 2009), and metabolomes (Aharoni et al., 2004; Fait et al., 2008; Zhang et al., 2011) from various *Fragaria* species.

Many excellent tools such as KAAS (Moriya et al., 2007), Mercator (May et al., 2008), MapMan (Thimm et al., 2004), RAST (Meyer et al., 2008; Wilke et al., 2016) etc. allow pathway-based (species-neutral) gene annotation analysis of omics data, but these may not provide information on the species-specific pathways, reactions, and metabolite variations and may not support the import/export of network data in the standardized formats. In contrast, FragariaCyc, based on BioCyc platform (supported by Pathway-Tools software), allows import and export of network data in the standard machine-readable SBML and BioPax formats and supports data interoperability (Jaiswal and Usadel, 2016). Due to the common underlying platform, exchange of relevant information between FragariaCyc and other BioCyc databases is greatly facilitated. Also, users can make quick cross-species comparisons of pathways, compounds, reactions, genes, and gene products with other publicly available BioCyc databases including AraCyc (*Arabidopsis thaliana*) (Mueller et al., 2003), RiceCyc (*Oryza sativa*) (Dharmawardhana et al., 2013), MedicCyc (*Medicago truncatul*a) (Urbanczyk-Wochniak and Sumner, 2007), MaizeCyc (*Zea mays*) (Monaco et al., 2013), PoplarCyc (*Populus trichocarpa*) (Zhang et al., 2010), VitisCyc (*Vitis vinifera*) (Naithani et al., 2014), etc. BioCyc databases also provide built-in tools for carrying out gene-expression analysis and flux balance analysis. Therefore, we chose the BioCyc platform to construct FragariaCyc.

Researchers can access various components of the FragariaCyc, and can conduct Omics Viewer analysis of expression data of their choice to assess the broader role of a gene(s), mutant and phenotype in the context of overall cellular metabolism. Here, publicly available transcriptomic data (Kang et al., 2013) was used to illustrate the functionality of Omics Viewer tool.

## Materials and methods

### Annotation of *F. vesca* proteins

FragariaCyc was developed based on the annotations of the version 1.0 hybrid gene models identified in the sequenced genome of the *F. vesca* L. ssp. *vesca* accession Hawaii 4 (https://www.rosaceae.org/species/fragaria/fragaria_vesca/genome_v1.0) (Shulaev et al., 2011). To enrich annotations of the *F. vesca* proteins, a workflow was employed to ascertain various conserved structural-functional domains, transmembrane domains and subcellular localization sequences. Subsequently, Gene Ontology (GO) annotations were imported for strawberry genes based on orthology to *Arabidopsis thaliana* from GO and TAIR (Poole, 2007) and *Oryza sativa* from Gramene (Tello-Ruiz et al., 2016). Using both methods, we provided preliminary annotations and GO assignments to 25,050 polypeptides. Similar methods have been employed earlier for the construction of RiceCyc (Dharmawardhana et al., 2013), MaizeCyc (Monaco et al., 2013), and VitisCyc (Naithani et al., 2014).

### Construction of FragariaCyc

The Pathway-Tools software (Karp et al., 2002) and the MetaCyc reference database (Caspi et al., 2014) were employed to create FragariaCyc by following standard protocols supplied by software developers. In the first step, following the PathoLogic input file format, a putative protein was assigned PRODUCT-TYPE code “P” and two required attributes, NAME and FUNCTION. The values for FUNCTION were supplied from structural-functional annotations and GO terms. Subsequently, optional attributes such as gene ID, gene symbols, SYNONYMS, EC number, GO assignments, and a free text COMMENT were assigned. Then GENETIC ELEMENT (required) and FASTA sequence files (optional) were added. In the second step, the “PathoLogic” option was performed with taxonomic filtering ON to find the best matches of enzyme name, gene name and synonyms, EC numbers, reactions, and pathways in the reference database MetaCyc. Once a correspondence was detected for a given entity associated with a reaction of the known pathway, the respective pathway was created in the FragariaCyc. Following the initial assembly of FragariaCyc, standard quality control checks were performed using a built-in consistency tool (Karp et al., 2002) and then plant-specific pathways were enriched by comparing it with PlantCyc (http://www.plantcyc.org/).

### Curation and updates of FragariaCyc

Several pathways and reactions specific to bacteria, fungi, and animals that bypassed the routine quality checks were manually removed from the FragariaCyc. Standard methods were employed to curate strawberry-specific pathways, reactions, metabolites and small molecules, genes, and literature citations. We have successfully incorporated the gene annotations based on recently published transcriptomic studies in *F. vesca* (Darwish et al., 2013, 2015; Kang et al., 2013). We continue to add and revise gene models in FragariaCyc based on the revised annotation of *F. vesca* Genome v1.1 (Darwish et al., 2015). The Pathway-Tool software and FragariaCyc contents are regularly updated. The current version of FragariaCyc, version 2.19, is hosted on the Pathway-Tool version 19.0.

### Omics viewer tool and gene expression analysis

The Omics Viewer tool within FragariaCyc allows analysis of large-scale expression data. To show the functionality of this tool, we analyzed a subset of publicly available RNA-Seq expression data (Kang et al., 2013) from *F. vesca* cultivar YW5AF7. The data file (Supplementary Table 1) was uploaded to the Omics Viewer tool for visualizing the differentially expressed genes on the “Cellular Overview Diagram,” and to understand the tissue-specific regulation of metabolic pathway genes.

## Results

### An overview of FragariaCyc, the cellular metabolic networks of *F. vesca*

Several species-specific plant metabolic pathway databases including FragariaCyc are available online from our website (http://pathways.cgrb.oregonstate.edu). At present, FragariaCyc contains 66 super-pathways grouped into 488 unique pathways, 2348 enzymatic and 101 transport reactions, 3507 enzymes, 289 transporters, and 2134 metabolites and small molecules.

**Figure 1:**
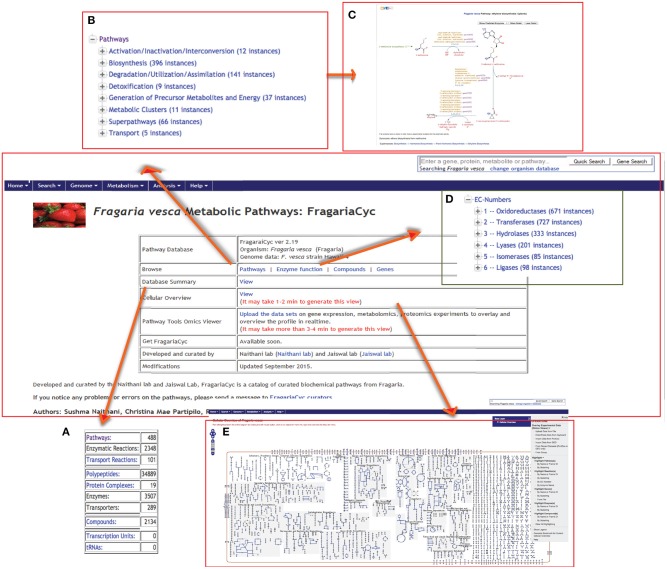
**A view of FragariaCyc homepage showing its features and functionalities, such as summary (A), browsing of pathways (B) and enzymes (D), a sample pathway page (C), and “Cellular Overview Diagram” (E)**. Users can also access Omics Viewer from this page to upload their data.

The FragariaCyc homepage provides hyperlinks to access a database summary, “Cellular Overview Diagram,” Omics Viewer. This homepage also facilitates “quick search” and browsing of the pathways, enzymes, genes and compounds using a hierarchical classification schema based on ontology (Figure 1). Every entity in the database has a detail page (e.g., ethylene biosynthesis pathway shown in Figure 1C) that provides a summary, literature citations, and other relevant information. “Cellular Overview Diagram” (a conceptual framework of the cellular metabolic networks) is also available from the pull-down “Metabolism” menu on the top navigation bar (Figure 1E).

**Figure 2:**
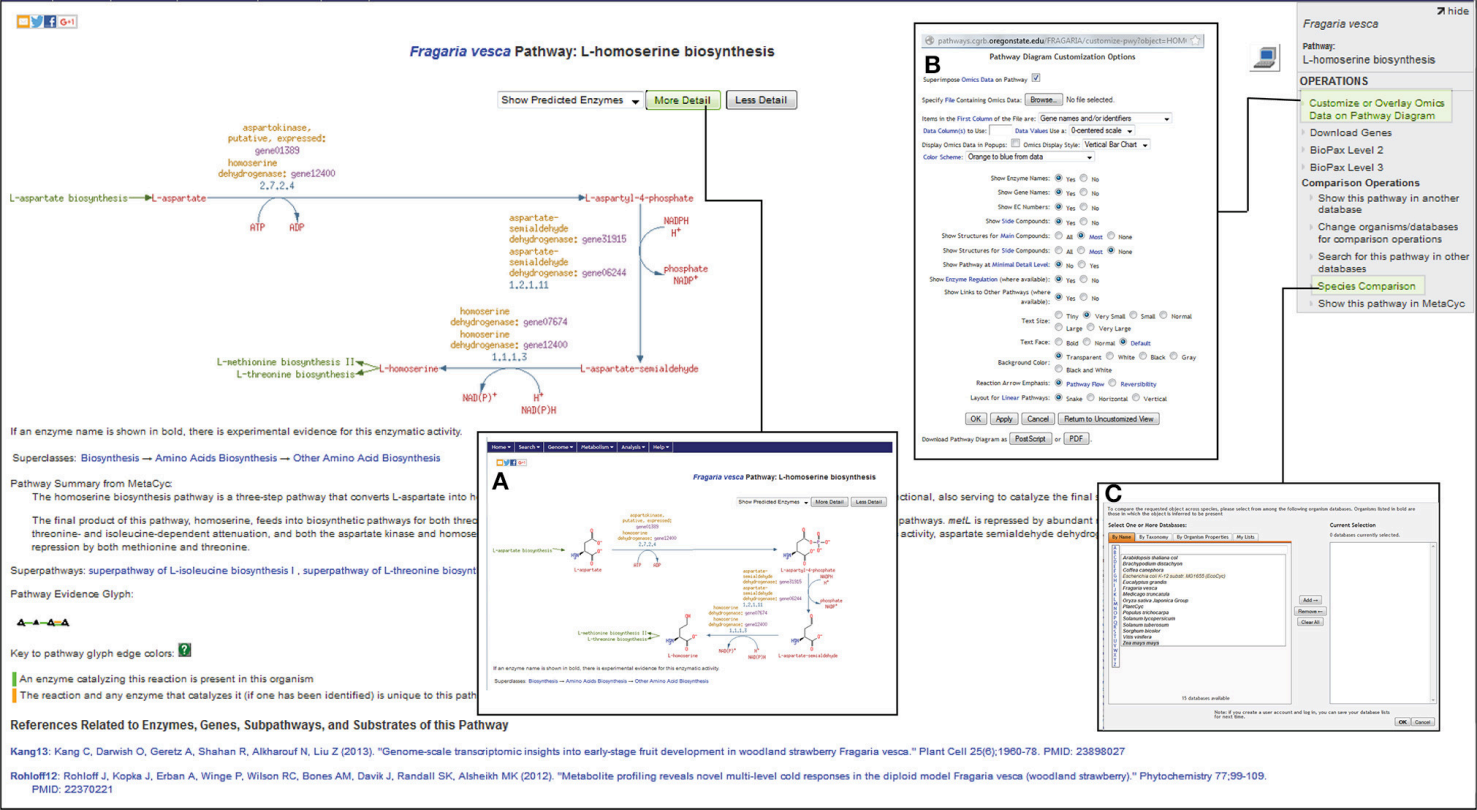
**A view of L-homoserine biosynthesis pathway page**. By clicking on more- or less-detail, users can manipulate the information shown on the page. For example, the most detailed output **(A)** shows the structures of the compounds. The links for the preceding L-aspartate biosynthesis pathway as well as for the successive biosynthesis pathways for L-methionine and L-threonine are highlighted in green color. The top right side panel shows links to Omics Viewer for uploading the expression data **(B)**, and for pathway comparisons across various species **(C)** by accessing other publicly available metabolic pathway databases from http://pathways.cgrb.oregonstate.edu.

Based on the ontology and enzyme function, pathways in FragariaCyc are organized into several categories: activation/inactivation/inter-conversion, biosynthesis, degradation/utilization/assimilation, detoxification, generation of precursor metabolites and energy, and transport. Typically, a pathway page provides a textbook style interactive diagram that includes hyperlinks to the detail pages of corresponding reactions, metabolites, enzymes, genes, cofactors, and small molecules. Figure 2 shows the L-homoserine biosynthesis pathway as an example of pathway detail page. The Pathway page also shows experimentally verified enzymes and associated genes in boldface. A right-hand side menu within a pathway page provides options to (i) upload expression data on a pathway diagram (Figure 2B); (ii) view a pathway in other plant species or reference database MetaCyc; and (iii) conduct pathway comparisons across two or more metabolic pathway databases (Figure 2C). For the convenience of researchers, our website hosts three reference databases, MetaCyc (Caspi et al., 2014), PlantCyc (Chae et al., 2012), and EcoCyc (Karp et al., 2000), and 13 species-specific plant pathway databases.

**Figure 3:**
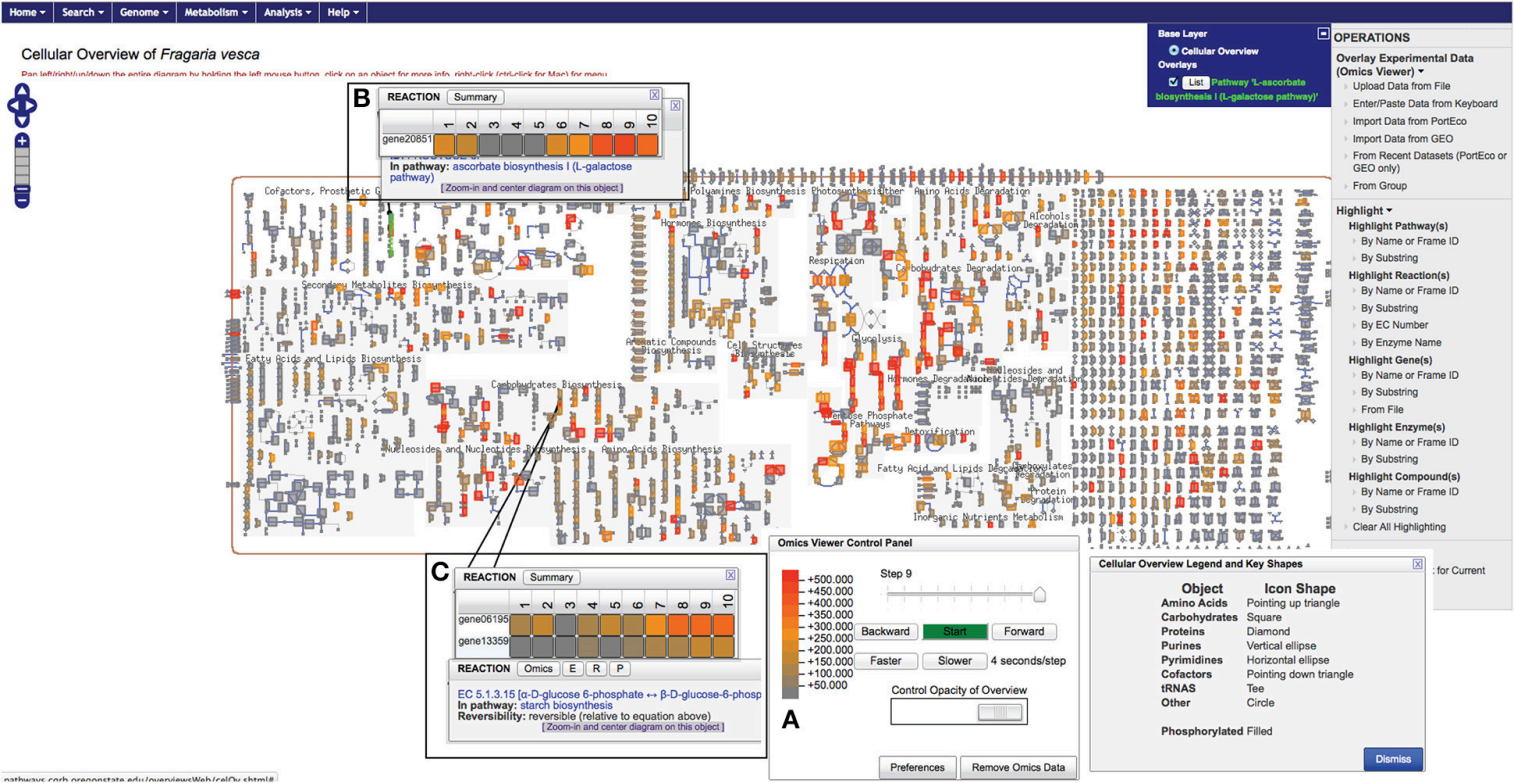
**A view of the *F. vesca* “Cellular Overview Diagram” displaying gene expression data from cortex4 tissue sample collected from small green berries**. The color scale used for depicting the expression level is shown **(A)**. The popup windows show the tissue-specific expression profile of a gene **(B)** or genes mapped to the same reaction **(C)**. Samples: #1, ovule1 (from open pre-fertilized flower); #2, ovule2 (achene collected just after fertilization containing a globular stage embryo); #3, embryo3 (heart-stage embryo from very small green berry); #4, embryo4 (torpedo-stage embryo from small green berry); #5, embryo5 (mature embryo with two cotyledons from big green berry); #6, cortex1 (from pre-fertilized flower); #7, cortex2 (from pollinated flower, 2–4 DPA); #8, cortex3 (from very small green berry); #9, cortex4 (from small green berry); #10, cortex5 (from big green berry) (Kang et al., 2013).

The “Cellular Overview Diagram” is accessible from the main page (Figure 1E). It represents a conceptual framework for metabolic networks and its various entities within a simple, non-compartmentalized *F. vesca* cell containing various specialized clusters of related pathways: biosynthesis pathways of primary metabolites, secondary metabolites, and co-factors; catabolic pathways; glycolysis and the TCA cycle; photosynthesis and energy-related pathways, etc. (Figure 3). In general, nodes of various shapes (for example, circle, diamonds, square, etc.) depict metabolites and lines depict either a metabolic or a transport reaction. Users can zoom into detailed view (by clicking on the widget located on the top left-hand side corner) to see the names of metabolites, reactions and associated enzymes with corresponding EC numbers (Figure 3). Typically, reactions that have not yet been assigned to a pathway represent the clutter at the extreme right side of the “Cellular Overview Diagram.” Additional options such as highlighting chosen entities and uploading of user-defined data on the “Cellular Overview Diagram” are available under the “OPERATIONS” menu on the right-hand side of the page (Figure 3).

### Manual curation of FragariaCyc

We constructed a strawberry metabolic network based on extensive computational analysis, automated and manual curation. Manual curation includes (i) confirmation of computational mappings of genes to reactions, and pathways; (ii) addition/editing of strawberry-specific compounds, reactions, sub-pathways, pathways and super-pathways including assignment of genes to the relevant enzyme and pathways based on gene orthology and from published studies; and (iii) deletion of nonplant pathways.

We added 773 compounds manually to FragariaCyc based on literature survey (Aharoni et al., 2004; Lunkenbein et al., 2006a; Fait et al., 2008; Osorio et al., 2011; Zhang et al., 2011; Rohloff et al., 2012; Kim et al., 2013; Gunduz and Ozdemir, 2014; Najda et al., 2014; Schwieterman et al., 2014; Voca et al., 2014; Xu et al., 2014). At present ~50% of the compounds out of the total 2069 are supported by experimental evidences. Many of these compounds impart high nutritional value to strawberries, such as flavonols, anthocyanins, and their derivatives. We have extensively edited biosynthesis pathways of several secondary metabolites including plant hormones (e.g., auxin, abscisic acid, brassinosteroids, ethylene, gibberellins and gibberellin precursors, jasmonic acid, etc.), phenylpropanoids, trans-lycopene, pro-anthocyanidins, isoflavonoids, zeaxanthin, beta-carotene, plant sterols, chlorophyllide *a*, and mevalonate.

Recently, Darwish et al. (2015) have revised annotation of *F. vesca* genome v1.1 based on transcriptomes of 25 different tissues in combination with the MAKER2 annotation pipeline (https://www.rosaceae.org/species/fragaria_vesca/genome_v1.1.a2) that identified 2286 new gene models, and 6006 new exons. The efforts to integrate these new genes and revised gene models into FragariaCyc are ongoing. However, we have updated annotations for 6598 genes based on the recent transcriptomics studies from *F. vesca* (Darwish et al., 2013, 2015; Kang et al., 2013).

We have not curated transport reactions in FragariaCyc so far, but FragariaCyc contains 101 transport reactions based on the automated baseline projections from MetaCyc.

### Omics viewer analysis of RNA-Seq data suggests tissue-specific regulation of metabolic pathways

The Omics Viewer tool enables users to upload and analyze a wide array of expression data (e.g., transcriptomes, proteomes, metabolomes, reaction flux data, etc.). We chose a subset of RNA-Seq expression data from the *F. vesca* line YW5AF7 (Kang et al., 2013) representing the differential expression of 16,316 genes during berry development in three tissues, ovule, vegetative cortex, and embryo (see Supplementary Table 1).

**Figure 4:**
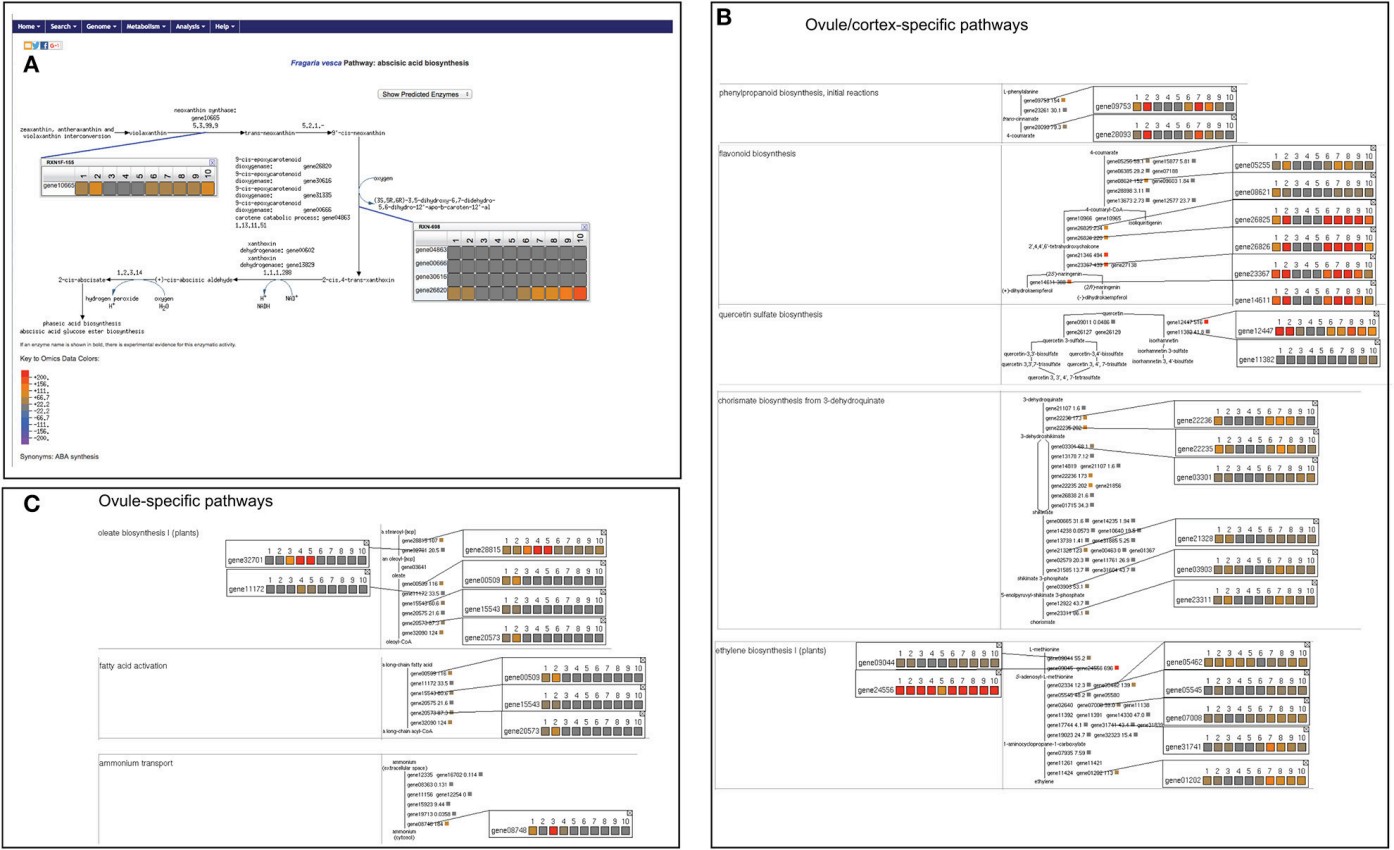
**Visualization of differentially expressed metabolic pathways. (A)** Abscisic acid biosynthesis pathway displaying expression profile of various associated genes. **(B)** A table of ovule or cortex-specific pathways with expression profiles of associated genes. **(C)** Ovule-specific pathways. By clicking on the genes and compounds more information can be accessed. The samples refer as #1, ovule1; #2, ovule2; #3, embryo3; #4, embryo4; #5, embryo5; #6, cortex1; #7, cortex2; #8, cortex3; #9, cortex4; #10, cortex5 (for details see Figure 3 legend). The common color scale shown in **(A)** depicts the expression level of genes and also applies to **(B,C)**.

We were able to map 16,271 out of the total 16,316 genes on FragariaCyc. The remaining 45 genes belong to newly identified *F. vesca* genes (Darwish et al., 2015) awaiting their addition into the database. We successfully mapped 2378 genes to the metabolic pathways and compared their expression across various samples. Figure 3A shows a “Cellular Overview Diagram” displaying the transcriptome of cortex4 (collected from stage-4 small green berry, cutoff value = 500). The “Cellular Overview Diagram” is an interactive platform, where users can click on reactions, compounds, and pathways to access more information about a particular entity and to navigate to respective detail pages. For example, a click on the initial reaction of the ascorbate biosynthesis I pathway opens a popup window showing the differential expression of gene20851 in samples #1-#10 (Figure 3B). Similarly, users can display expression profiles of the gene homologs (Figure 3C) that map to the same enzyme. It is evident from the expression profiles of several gene-duplicates that homologous genes are likely to respond to different signals or regulators. Additional options from the right-hand side panel allow highlighting pathways/reactions/genes within the “Cellular Overview.” Users can also use the pathway customization tool to import expression data onto desired pathway pages. For example, Figure 4A shows the mapping of transcriptomic data on the abscisic acid biosynthesis pathway.

Furthermore, we generated a table of differentially regulated pathways (cutoff = 200) to identify ovule-, embryo-, and cortex-specific pathways. As shown in Figure 4, enzymes involved in the biosynthesis pathways of ethylene, 4-coumerate, flavonoids, chorismate, and quercetin are highly expressed in the cortex and/or in ovule1 and ovule2, but are clearly repressed in the embryo. In contrast, several genes of oleate biosynthesis show preferential expression in ovule1 and ovule2, and some expression in the developing embryo, but clearly repressed in the cortex (Figure 4C). For example, genes involved in fatty acid activation are clearly repressed in embryo and cortex, but express in the ovule. Another gene encoding an ammonium transporter expresses in the ovule and embryo, but not in the cortex (Figure 4C).

We investigated changes in the expression of genes involved in the biosynthesis of various plant hormones during the early fruit development in embryo and cortex, and pre- and post-fertilized ovules. In general, auxin biosynthesis genes show the highest expression in the tissues derived from small green berries and then slowly decline as the berry grows. Many embryo-specific genes involved in indole-3-acetate biosynthesis I, such as gene11728 (encoding indole-3-pyruvate monooxygenase), gene03586 (encoding L-tryptophan aminotransferase), gene04251 (encoding carboxylesterase), and gene22572 (encoding an amidotransferase) show the highest expression embroy4 tissue (from small green berries) and then show a decline in their expression in embryo5 (from big green berry). Similarly, other auxin biosynthesis pathway genes expressed in the cortex and ovules showed higher expression in stage-4 small green berry and decreased expression in stage-5 big green berry. Gibberellins (GA) biosynthesis genes (e.g., gene19437, gene13360, gene01059, etc.) show higher expression in embryo following fertilization event. Low expression of a few GA biosynthesis genes was observed in the cortex tissue as well. In general, no significant change in the expression of GA biosynthesis gene was observed between the samples obtained from small and big green berries (Supplementary Figure 1B).

The abscisic acid biosynthesis genes show a gradual increase in their expression during berry development in the cortex tissue, eventually attaining a peak at stages 4–5 (Figure 4A). However, no significant change or very little increase in their expression was observed in the embryos. The genes involved in initial steps of ethylene biosynthesis show higher expression across all tissue types. However, gene01202 and gene07935 (encoding for the enzyme 1-aminocyclopropane -1-carboxylate oxidase catalyzing the final step) show higher expression in the cortex, compared to the embryo (Figure 4B). The majority of jasmonic acid biosynthesis genes show preferential expression in the cortex throughout the early stages of berry development, although gene16355, gene28898, and gene16355 show embryo-specific expression (Supplementary Figure 1C). The majority of brassinosteroid biosynthesis genes show high expression in the ovule, embryo and cortex tissues (Supplementary Figure 1D), although a few genes show highly tissue-specific expression profile (e.g., gene20962, gene19221, gene03747).

Overall, the Omics Viewer analysis can reveal information about subcellular/cell-/tissue-/organ-specific localization of a metabolic pathway; differential expression of gene family members mapped to the same reaction; and help to identify the rate-limiting step within a pathway—all required for the identification of candidate genes for the purpose of cultivars improvement.

## Discussion

To date, FragariaCyc is the only resource that provides a comprehensive knowledgebase of strawberry metabolism supported by extensive manual curation and allows analysis of omics data in the context of the cellular-level metabolic network. FragariaCyc is based on the sequenced genome of *F. vesca* (Shulaev et al., 2011) and shares functionalities with other BioCyc pathway databases including several species-specific plant pathway databases (Mueller et al., 2003; Urbanczyk-Wochniak and Sumner, 2007; Dharmawardhana et al., 2013; Monaco et al., 2013; Naithani et al., 2014).

The published literature and publicly available experimental data serve as a vital resource for improving the functional annotations and curation of the genes, enzymes, reactions, pathways, and compounds in FragariaCyc. We have updated gene annotations in FragariaCyc using information from several published studies (Aharoni et al., 2002; Wein et al., 2002; Bianco et al., 2009; Osorio et al., 2011; Darwish et al., 2013, 2015; Kang et al., 2013; Xu et al., 2014). In 2011, an improved version of the *F. vesca* genome assembly became available (https://www.rosaceae.org/species/fragaria/fragaria_vesca/genome_v1.1), but no new genes were predicted. Recently, a large-scale transcriptomic study (Darwish et al., 2015) identified 2286 new genes and 6006 new exons (by correcting the exon-intron boundaries in several genes). Our efforts to incorporate newly identified and revised genes in FragariaCyc, and their mapping to appropriate reactions and pathways are ongoing. We expect that FragariaCyc as a community resource will continue to mature as more experimental data become available and integrated into it.

We anticipate that FragariaCyc will aid strawberry researchers in (i) discovering the broader role of new and known genes/molecular interactions in the context of overall cellular metabolism; (ii) understanding how a plant's environment and genetic diversity relates to yields and the quality of its fruit (e.g., texture, taste, aroma, flavor, etc.); and (iii) exploring how the plant's overall metabolism and its ability to adapt are affected in response to climate change. To show the utility of the Omics Viewer, we analyzed a subset of publicly available RNA-Seq data from *F. vesca* (Kang et al., 2013) to understand tissue-specific regulation of the metabolic pathways during fruit development.

Figure 3 shows visualization of RNA-Seq data over “Cellular Overview Diagram.” Users can customize the display of expression data by defining a cut-off value, and/or choosing an available color schema to depict up- and down-regulation of the genes. When data from multiple samples are uploaded, an automated animation displays painted Cellular Overview Diagrams in sequential order. Users have options to capture this animation as a movie (by using a third-party software) or to pause it at desired sample (e.g., Figure 3) for further exploration. The users can also display expression data on the pathway diagram, and generate a table of pathways in which one or more genes show differential expression (Figure 4).

The unique feature of Omics Viewer analysis is its ability to compare expression profiles of paralogs mapped to the same reaction considering extensive gene duplications in plant genomes (Shiu and Bleecker, 2003; Jaillon et al., 2007; Velasco et al., 2007; Myburg et al., 2014; Renny-Byfield and Wendel, 2014). The information about differential expression of the homologous genes can help researchers to articulate functional redundancies. Such analysis is very useful for characterizing a mutant/phenotype, and for shortlisting candidate genes for functional characterization using transient (Guidarelli and Baraldi, 2015) or stable transformation systems available for strawberry (Slovin et al., 2009). Also, the mapping of the expression profile of genes in a given pathway can identify (i) a rate limiting step in a pathway, and (ii) clusters of pathways regulated in response to a common signal/regulator.

Strawberry is not a true fruit. Indeed, the fleshy edible fruit, known as “receptacle,” develops from the stem tip instead of an ovary. The receptacle is the primary site of accumulation of various nutrients and metabolites (Fait et al., 2008; Bianco et al., 2009) that impart color, flavor and taste to the berry. The receptacle is made of two tissues: the fleshy tissue placed immediately underneath achenes is called the cortex, and the interior tissue is known as the pith. The seed-like achenes, embedded in the fleshy tissue (receptacle) are the real fruit as they develop from an ovary and contain developing embryo inside. Thus, strawberry offers an interesting system to explore how various accessory fruits relate to each other, and to the true fleshy fruits (e.g., grape and tomato) regarding their global gene expression profile, cell signaling, and metabolic milieu.

Overall, our analysis suggests that the majority of auxin and gibberellin biosynthesis genes show preferential expression in the embryo, which is consistent with their role in driving early fruit development (Nitsch, 1950; Dreher and Poovaiah, 1982; Csukasi et al., 2011; Kang et al., 2013). The levels of free auxins in both the achene and the receptacle have been shown to rise after fertilization, peak at small green fruit stage, and then decline slowly in big green fruit (Dreher and Poovaiah, 1982; Symons et al., 2012).

In contrast, genes involved in the biosynthesis of the abscisic acid, ethylene, and jasmonic acid show higher expression in the cortex tissue when compared with embryos, and their expression peaks at the stage-4 small green or stage-5 big green berries. Abscisic acid has been shown to play a key role in the ripening of strawberry (Jia et al., 2011; Li et al., 2011; Vallarino et al., 2015) and tomato (Zhang et al., 2009), likely by triggering ethylene biosynthesis. Ethylene is known as a major driver of ripening in climacteric fruits, such as apple and tomato (Seymour et al., 2013). However, the interplay between various hormones is more likely to foster ripening transition in non-climacteric fruits, such as strawberry (Kang et al., 2013; Mcatee et al., 2013; Osorio et al., 2013; Fortes et al., 2015). Recent studies suggest some role of ethylene in non-climacteric fruit ripening in both grapes and strawberries (Merchante et al., 2013; Lopes et al., 2015). Brassinosteroids have also been proposed to positively influence ripening associated processes, whereas, jasmonic acid seems to interfere with the ripening (Jia et al., 2011; Concha et al., 2013; Merchante et al., 2013). The large-scale gene expression studies suggest similarities at the molecular level between climacteric and non-climacteric ripening process. However, the relative contribution of various hormones in fruit ripening could vary between two categories.

FragariaCyc is freely accessible online at http://pathways.cgrb.oregonstate.edu. The data content in FragariaCyc will updated once a year as long as we have some support for curation. Afterward, an archive copy of FragariaCyc will be maintained on our website and will be deposited in a public repository. Users are encouraged to view an online video on metabolic pathway databases available at http://biocyc.org/webinar.shtml.

## Author contributions

SN and PJ conceived the project. SN led the project and its overall curation process. SN and CP did curation. PJ and RR constructed the automated projections of the FragariaCyc. JE carried out the Inparanoid-based annotations and provided technical support. SN analyzed RNA-Seq expression data. SN and PJ wrote the manuscript.

## Funding

This work was supported by the startup funds provided to SN and PJ by Oregon State University.

### Conflict of interest statement

The authors declare that the research was conducted in the absence of any commercial or financial relationships that could be construed as a potential conflict of interest.

## Supplementary material

Supplementary Table 1 **RNA-Seq expression data used for Omics Viewer analysis**. This is a subset of publicly available data from *F. vesca* (Kang et al., 2013) that represent differential expression of 16,316 genes across 10 samples: ovule1 and cortex1 were from open flower prior to fertilization, ovule2 and cortex2 are from pollinated flowers just after fertilization, while the cortex3-5 and embryo3-5 were collected from the developing strawberry as described previously (Kang et al., 2013).

Supplementary Figure 1 **Differential expression of genes using absolute RPKM values (cutoff = 50) associated with gibberellin (B), jasmonic acid (C), and brassinosteroid (D), biosynthesis pathway during fruit development in three tissues: ovule, embryo, and cortex**. A common color scale shown **(A)** depicts the expression level of genes belonging to pathways **(B–D)**. The samples refer as #1, ovule1; #2, ovule2; #3, embryo3; #4, embryo4; #5, embryo5; #6, cortex1; #7, cortex2; #8, cortex3; #9, cortex4; #10, cortex5 as described in Figure 3.
