# Supplementary figures and images for: FragariaCyc: A Metabolic Pathway Database for Woodland Strawberry Fragaria vesca
Source: Front Plant Sci. 2016 Mar 4;7:242. doi: 10.3389/fpls.2016.00242 (PMC4777718; doi:10.3389/fpls.2016.00242)

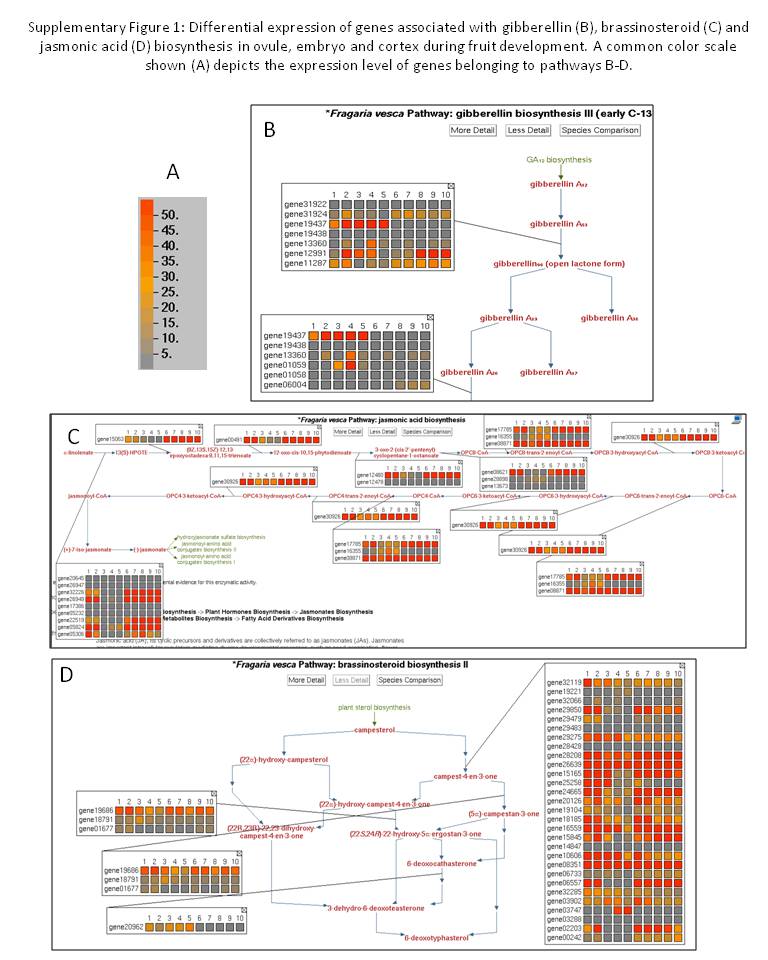

Supplement: Supplementary Figure 1 — Differential expression of genes using absolute RPKM values (cutoff = 50) associated with gibberellin (B), jasmonic acid (C), and brassinosteroid (D), biosynthesis pathway during fruit development in three tissues: ovule, embryo, and cortex. A common color scale shown (A) depicts the expression level of genes belonging to pathways (B–D). The samples refer as #1, ovule1; #2, ovule2; #3, embryo3; #4, embryo4; #5, embryo5; #6, cortex1; #7, cortex2; #8, cortex3; #9, cortex4; #10, cortex5 as described in Figure 3. [file Image1.jpg]
